# Supplementary material for: Global biogeography of microbes driving ocean ecological status under climate change
Source: Nat Commun. 2024 May 31;15:4657. doi: 10.1038/s41467-024-49124-0 (PMC11143227; doi:10.1038/s41467-024-49124-0)
Supplement: Supplementary file 1 — Supplementary Information [file 41467_2024_49124_MOESM1_ESM.pdf]

Supplementary Information for

Global biogeography of microbes driving ocean ecological status under climate change

Zhenyan Zhang[1,†], Qi Zhang[1,2,†], Bingfeng Chen[1], Yitian Yu[1], Tingzhang Wang[3], Nuohan Xu[1,2], Xiaoji Fan[3], Josep Penuelas[4,5], Zhengwei Fu[6], Ye Deng[7], Yong-Guan Zhu[7,8], Haifeng Qian[1,*]

1. College of Environment, Zhejiang University of Technology, Hangzhou 310032, P. R. of China
2. College of Chemistry & Chemical Engineering, Shaoxing University, Shaoxing 312000, P. R. of China
3. Key Laboratory of Microbial Technology and Bioinformatics of Zhejiang Province, Hangzhou 310012, P. R. of China
4. CSIC, Global Ecology Unit CREAF-CSIC-UAB, Bellaterra, Barcelona 08193, Catalonia, Spain
5. CREAF, Campus Universitat Autònoma de Barcelona, Cerdanyola del Vallès, Barcelona 08193, Catalonia, Spain
6. College of Biotechnology and Bioengineering, Zhejiang University of Technology, Hangzhou 310032, P. R. of China
7. State Key Laboratory of Urban and Regional Ecology, Research Center for Eco-environmental Sciences, Chinese Academy of Sciences, Beijing 100085, P. R. of China
8. Key Laboratory of Urban Environment and Health, Institute of Urban Environment, Chinese Academy of Sciences, Xiamen 361021, P. R. of China

[†]Zhenyan Zhang and Qi Zhang contributed equally to this work.

[*]Correspondence to: Haifeng Qian (hfqian@zjut.edu.cn)

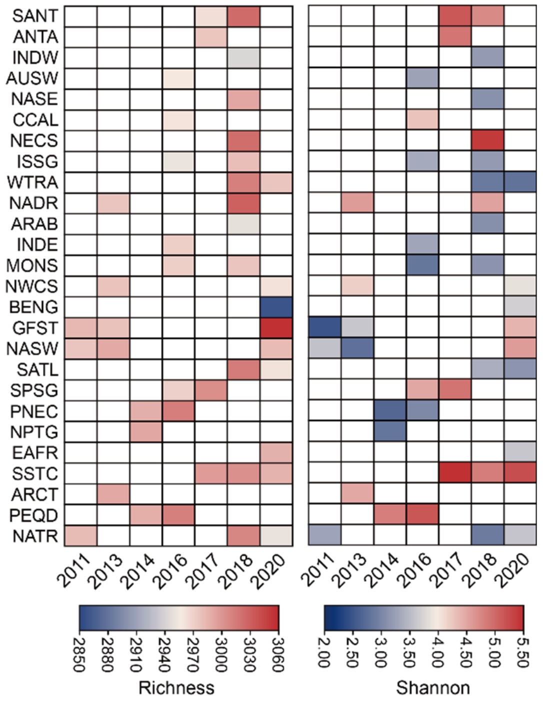

Supplementary Figure 1. Spatial and temporal variation in the diversity of microbial communities in the surface ocean. A detailed description of each Longhurst Province can be found in Supplementary Data 1.

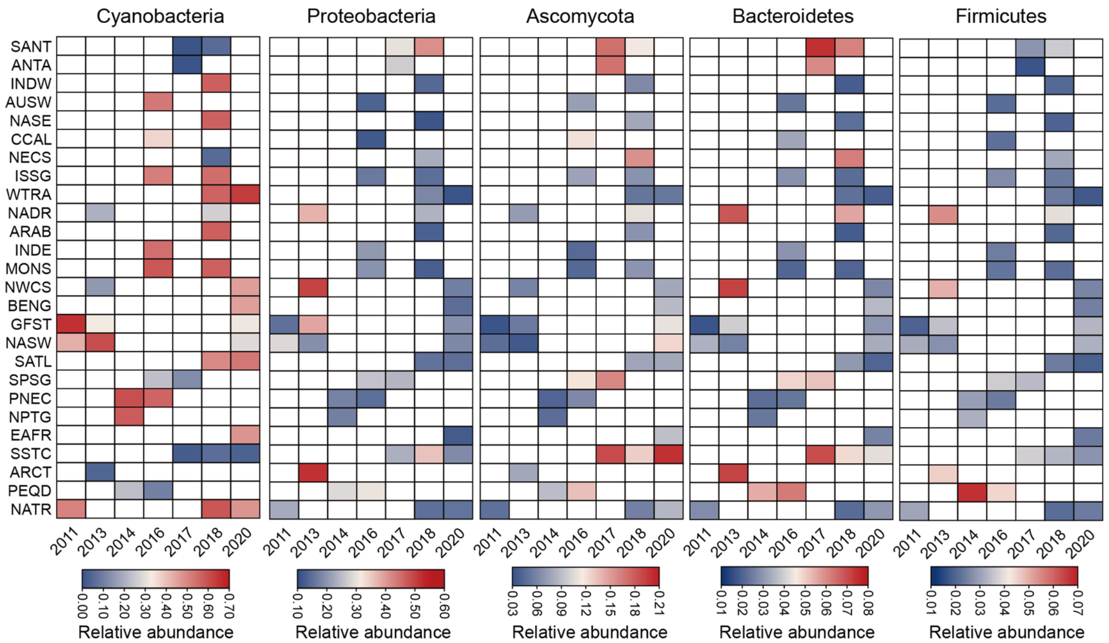

Supplementary Figure 2. Spatial and temporal variation in the structure of microbial communities in the surface ocean. A detailed description of each Longhurst Province can be found in Supplementary Data 1.

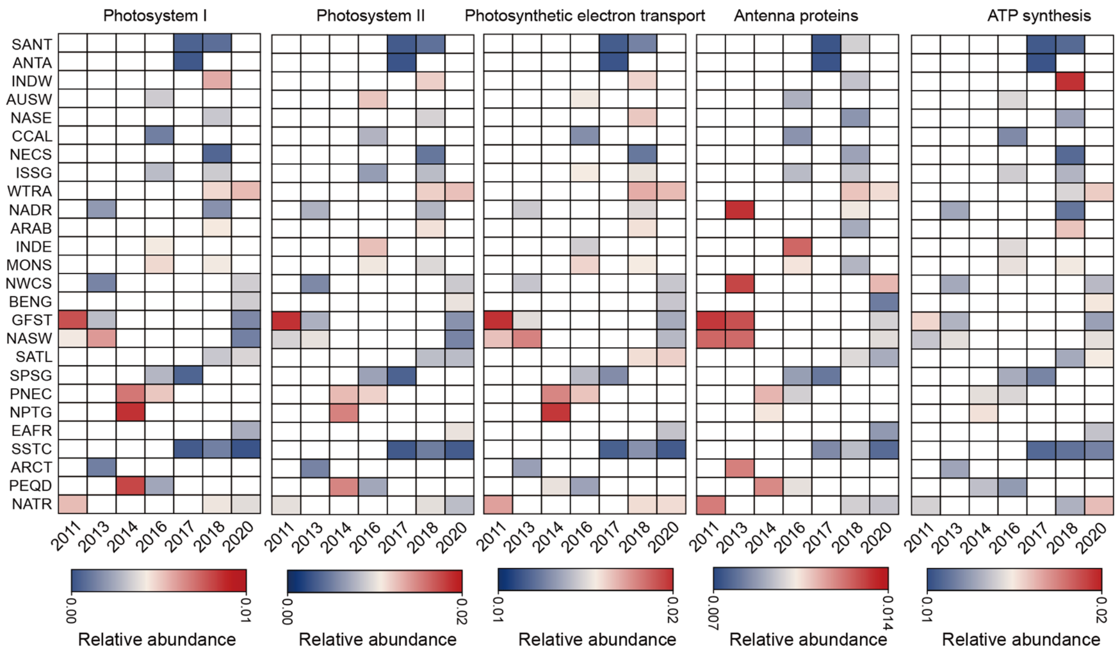

Supplementary Figure 3. Spatial and temporal variation in the photosynthetic potential of microbial communities in the surface ocean. A detailed description of each Longhurst Province can be found in Supplementary Data 1. ATP: adenosine triphosphate.

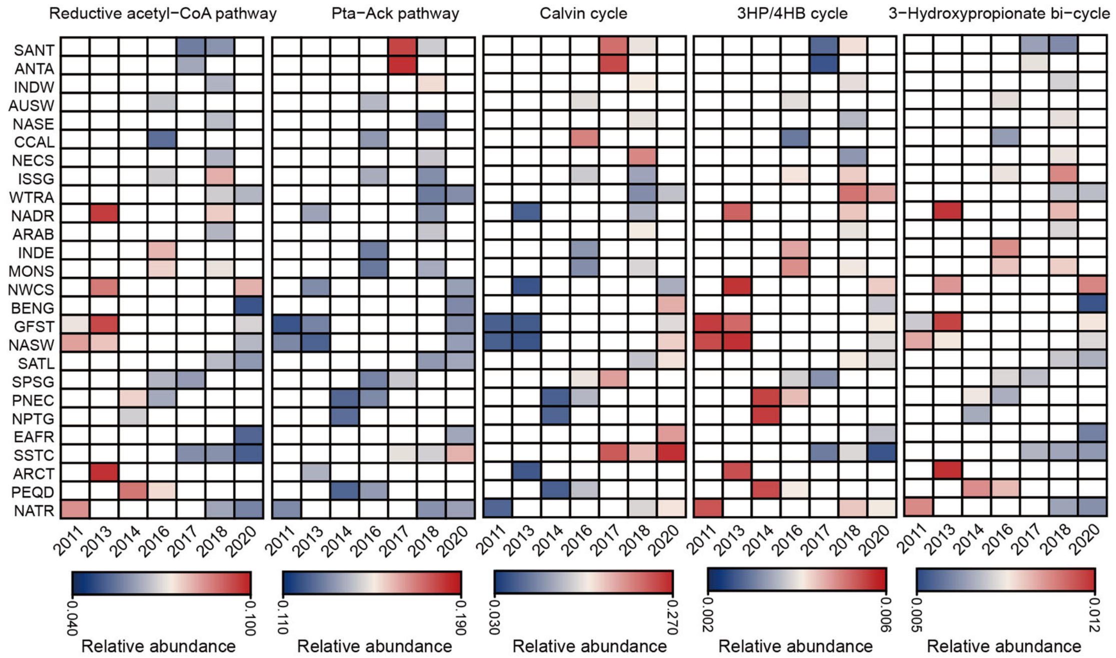

Supplementary Figure 4. Spatial and temporal variation in the carbon fixation potential of microbial communities in the surface ocean. Pta-Ack: Phosphate acetyltransferase-acetate kinase; 3HP/4HB: 3-hydroxypropionate/4-hydroxybutyrate. A detailed description of each Longhurst Province can be found in Supplementary Data 1.

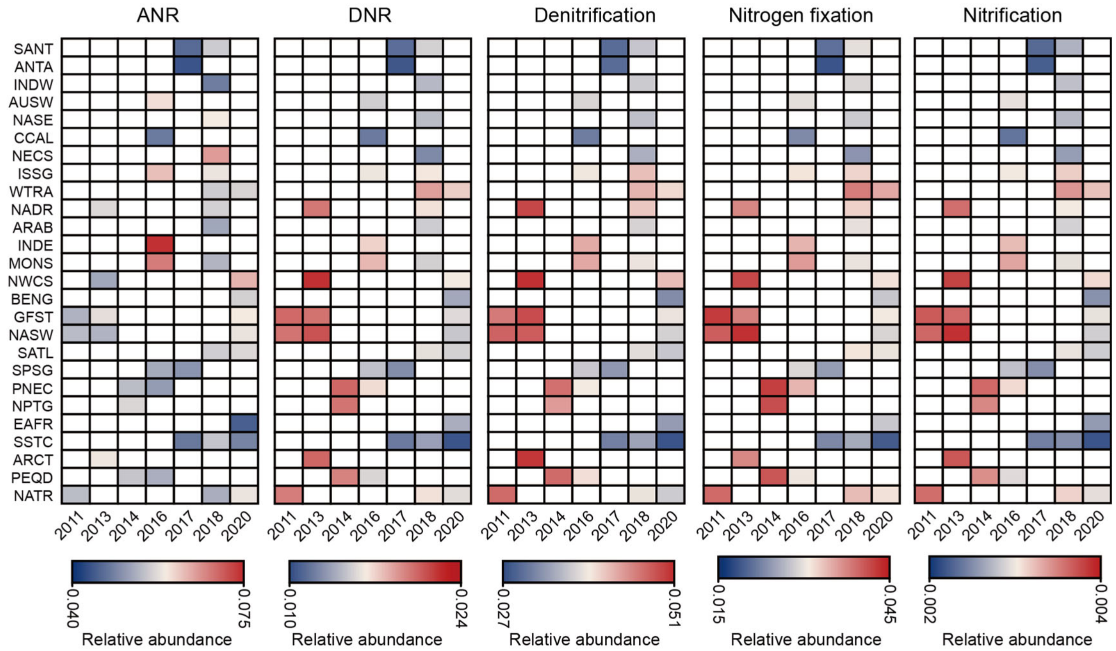

Supplementary Figure 5. Spatial and temporal variation in the nitrogen metabolism potential of microbial communities in the surface ocean. ANR: Assimilatory nitrate reduction; DNR: Dissimilatory nitrate reduction. A detailed description of each Longhurst Province can be found in Supplementary Data 1.

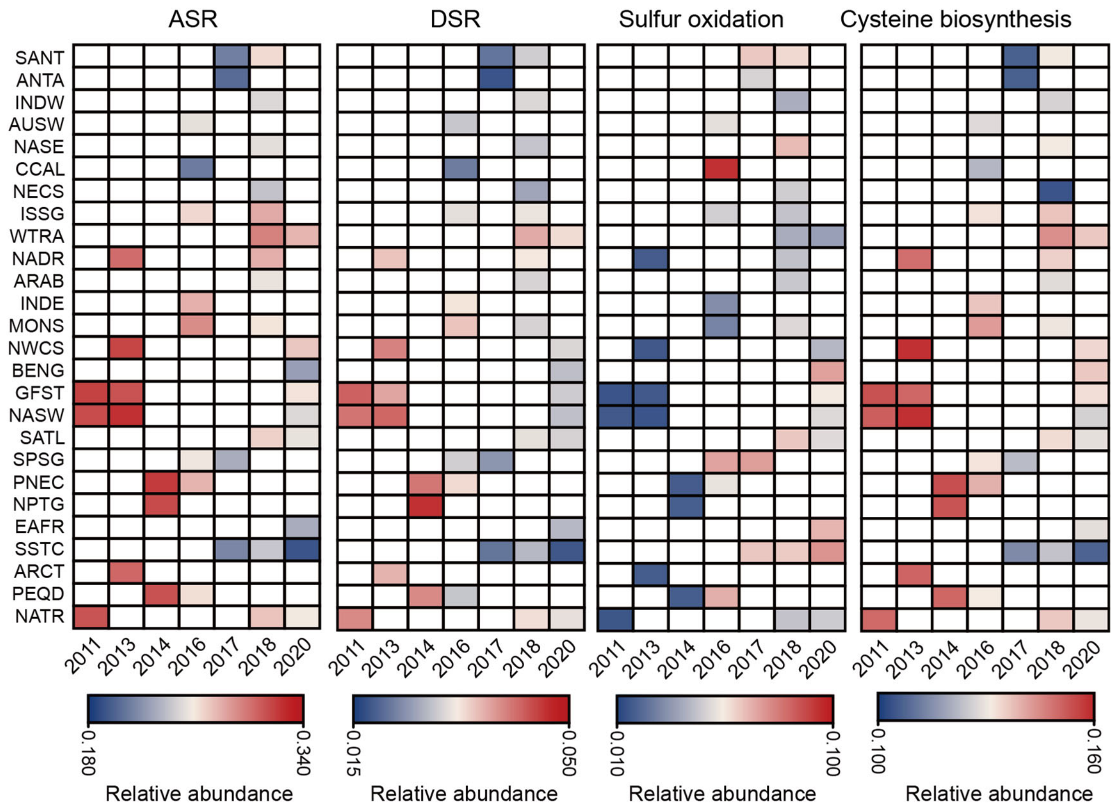

Supplementary Figure 6. Spatial and temporal variation in the sulfur metabolism potential of microbial communities in the surface ocean. ASR: Assimilatory sulfate reduction; DSR: Dissimilatory sulfate reduction. A detailed description of each Longhurst Province can be found in Supplementary Data 1.

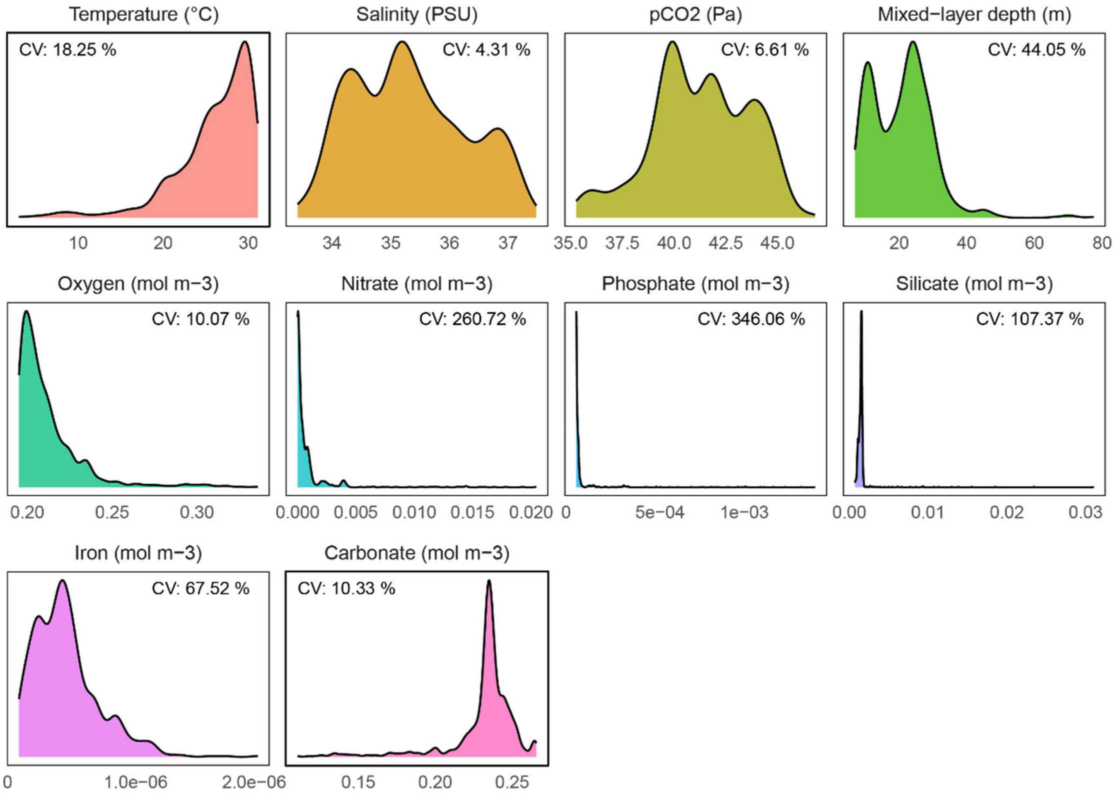

Supplementary Figure 7. Variations in ten environmental factors in the surface ocean. CV: coefficient of variation; PSU: practical salinity units.

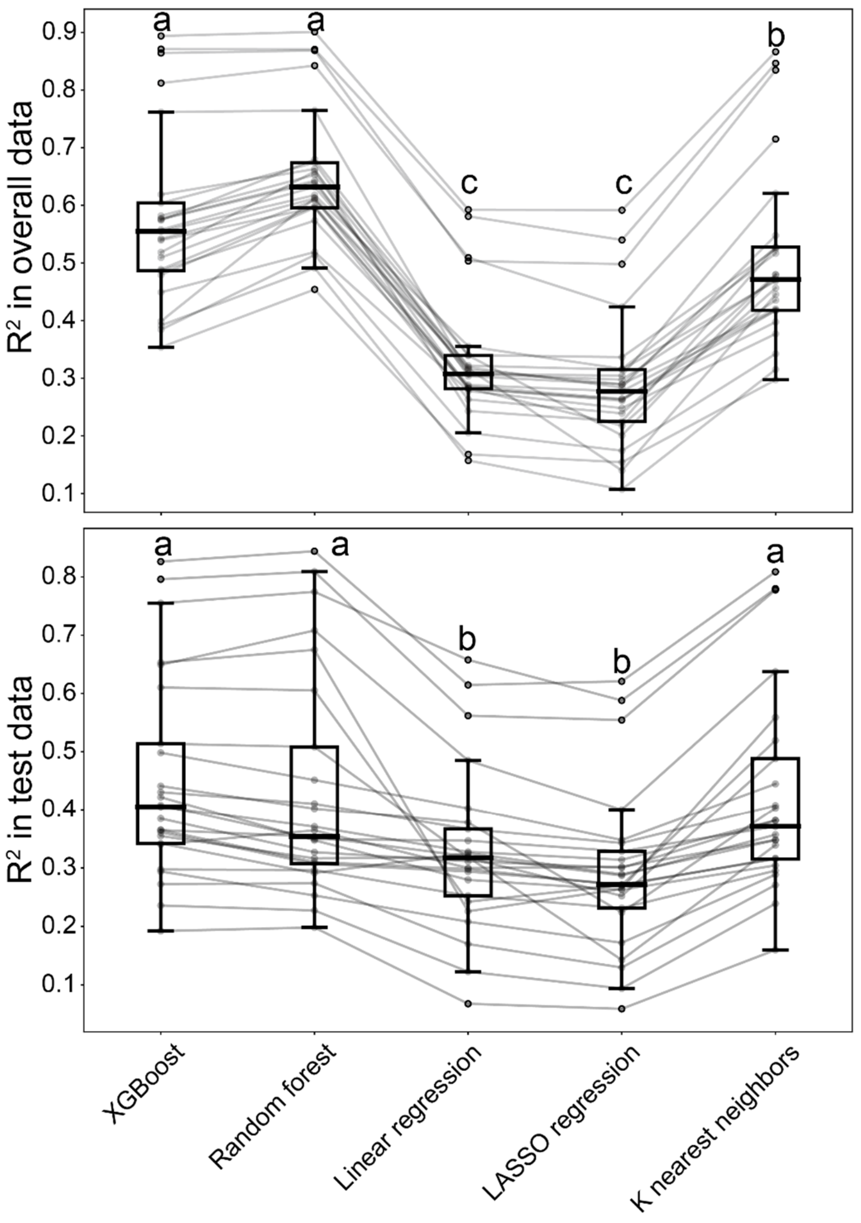

Supplementary Figure 8. Performance of machine learning models constructed by different algorithms under the best hyperparameter combinations. The random forest algorithm yielded the highest R^2 values for all samples (overall data, n = 890) and test samples (20% of all samples). Different letters (a, b, c) represent significant differences ($p < 0.05$) according to the Friedman test with Nemenyi pairwise comparisons (paired samples). XGBoost: eXtreme gradient boosting; LASSO regression: least absolute shrinkage and selection operator regression.

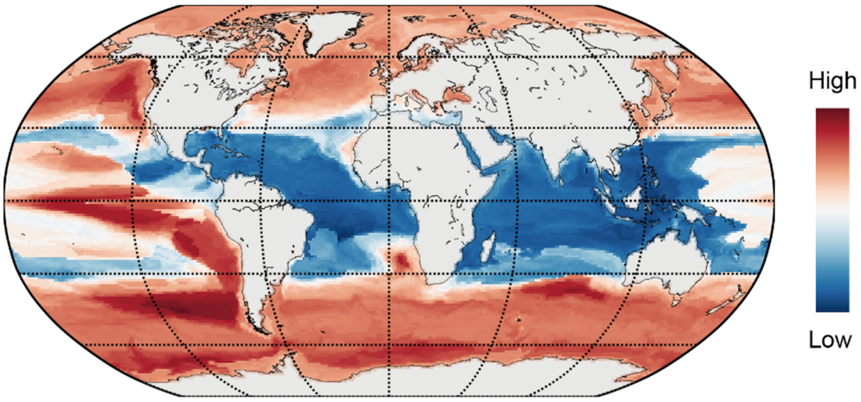

Supplementary Figure 9. Global distributions of the Shannon index of ocean microbial communities predicted under current environmental conditions (2023).

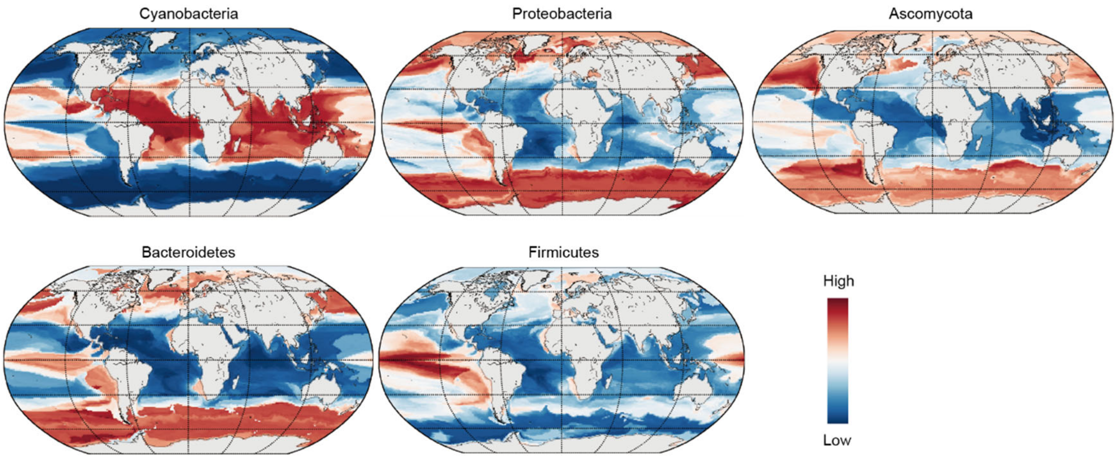

Supplementary Figure 10. Global distributions of dominant taxa in ocean microbial communities predicted under current environmental conditions (2023).

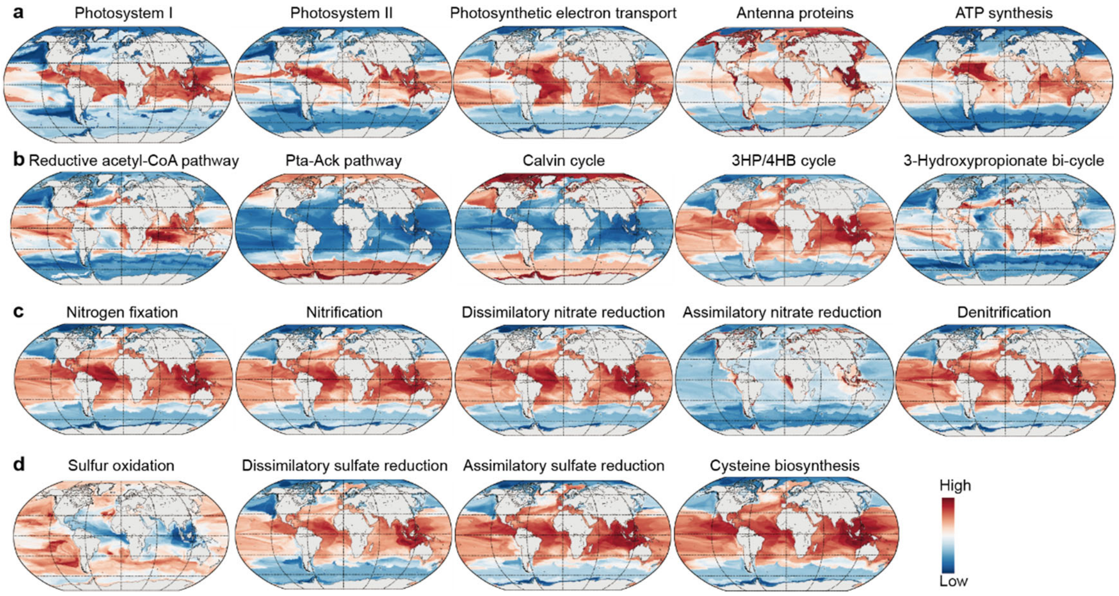

Supplementary Figure 11. Global distributions of biogeochemical potentials of ocean microbial communities predicted under current environmental conditions (2023). a, Pathways involved in photosynthesis; **b,** pathways involved in carbon fixation; **c,** pathways involved in nitrogen metabolism; **d,** pathways involved in sulfur metabolism. ATP: adenosine triphosphate; Pta-Ack: Phosphate acetyltransferase-acetate kinase; 3HP/4HB: 3-hydroxypropionate/4-hydroxybutyrate.

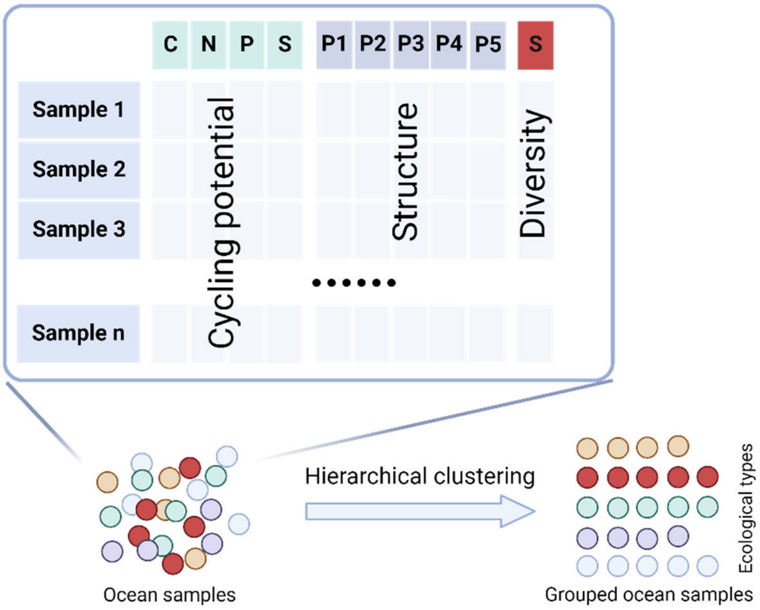

Supplementary Figure 12. Framework for clustering ocean samples according to ecological status by integrating the biogeochemical potential, structure, and diversity of microbial communities. This figure was visualized using BioRender (https://app.biorender.com/) under a Creative Commons Attribution-NonCommercial-NoDerivs 4.0 International license (https://creativecommons.org/licenses/by-nc-nd/4.0/legalcode.en).

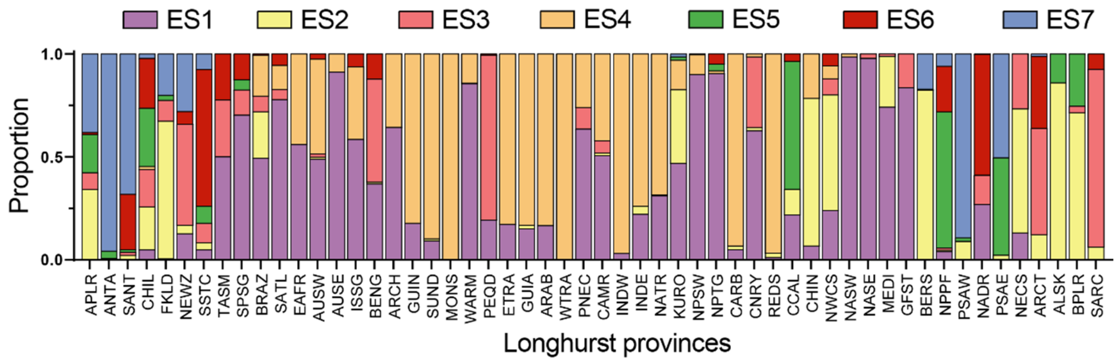

Supplementary Figure 13. Proportion of seven types of ecological status in each Longhurst Province in 2023. A detailed description of each Longhurst Province can be found in Supplementary Data 1.

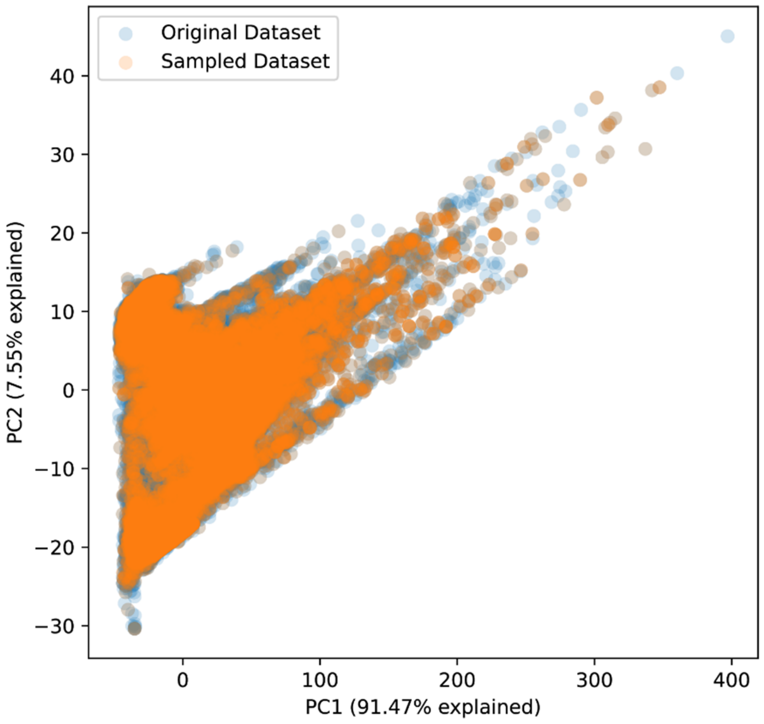

Supplementary Figure 14. Composition of environmental factors in the original dataset and sampled dataset.

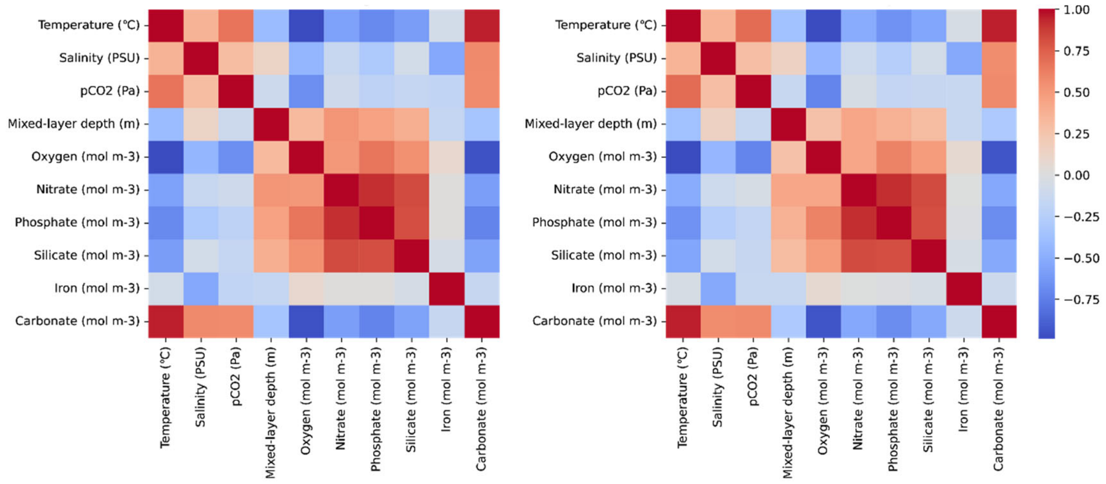

Supplementary Figure 15. Correlations between environmental factors in the original dataset and the sampled dataset. Heatmap exhibited the Pearson's correlation. PSU: Practical salinity units.

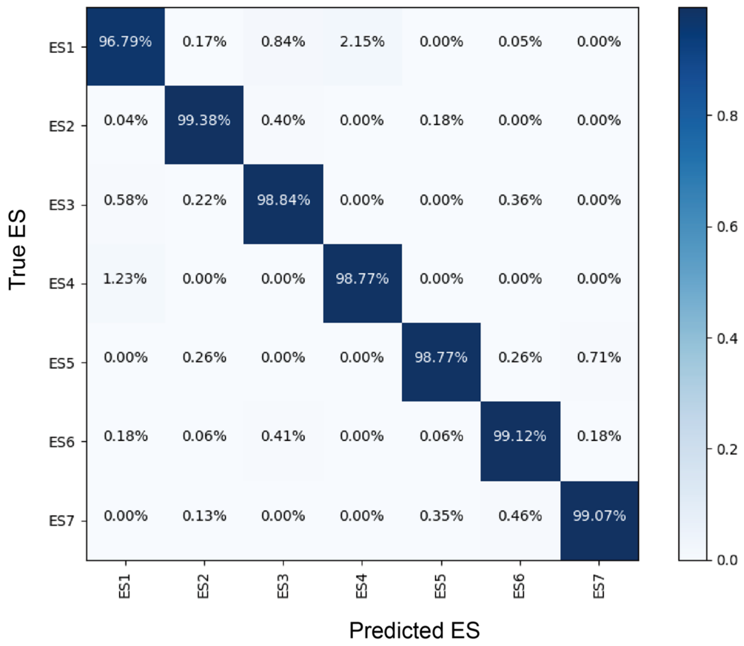

Supplementary Figure 16. The confusion matrix confirmed the good performance of the final model in the classification of ecological status. Heatmap exhibited the proportion of each predicted type of ecological status in each true type of ecological status.

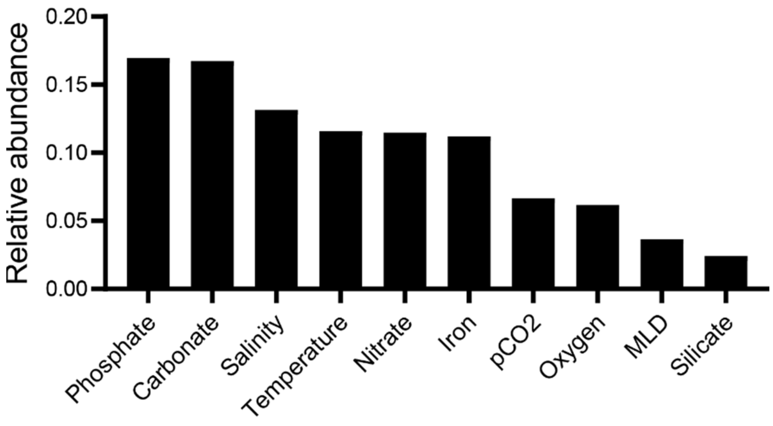

Supplementary Figure 17. The relative importance of different environmental factors in the classification of ecological status. MLD: mixed-layer depth.

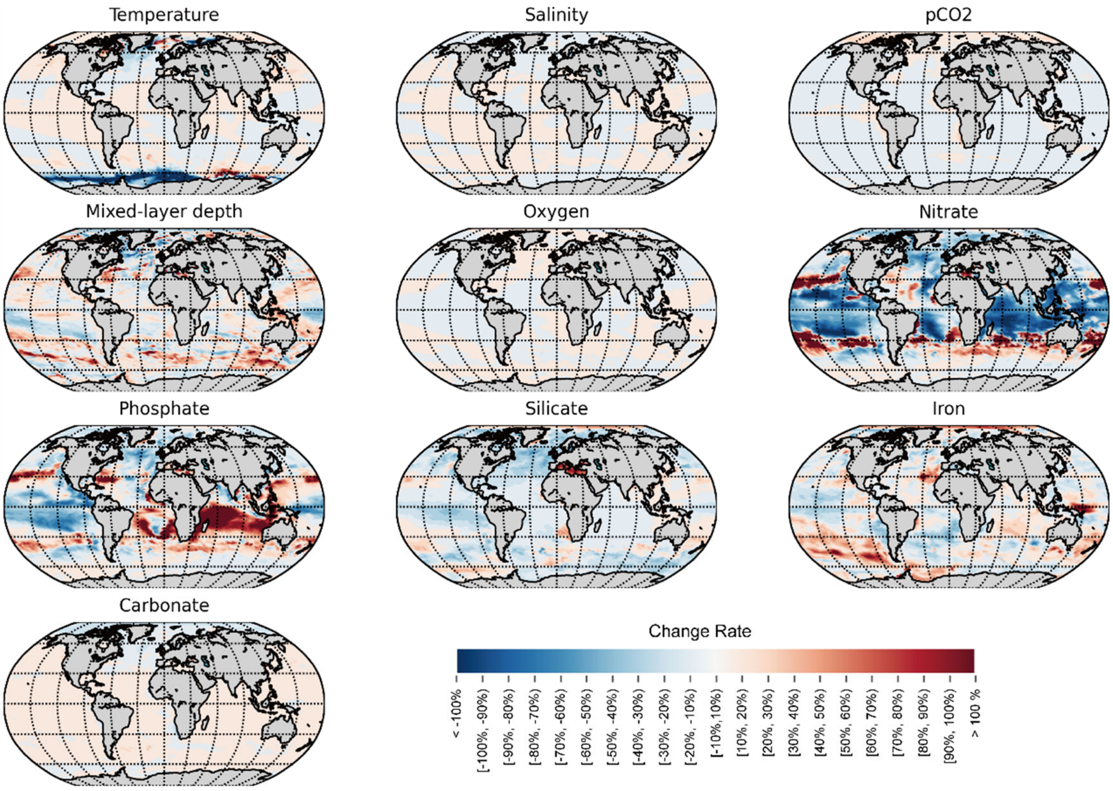

Supplementary Figure 18. Changes in environmental factors in 2100, compared to those in 2023, under the SSP1-1.9 scenario.

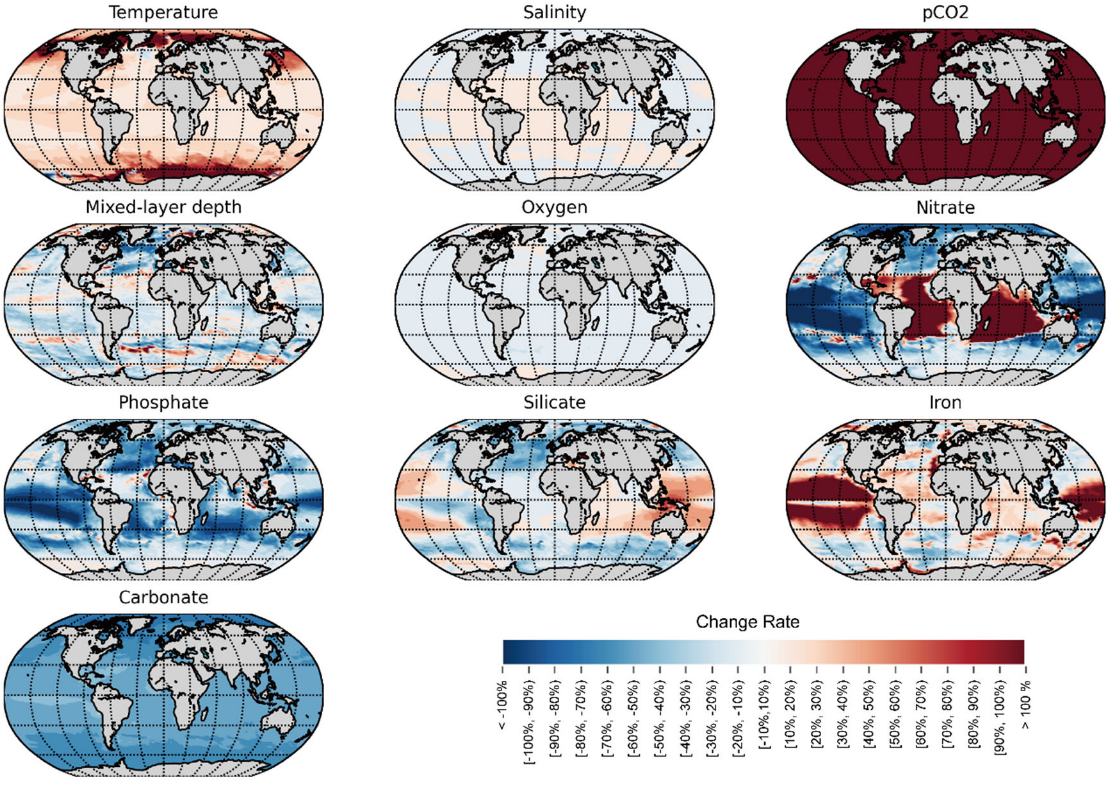

Supplementary Figure 19. Changes in environmental factors in 2100, compared to those in 2023, under the SSP5-8.5 scenario.

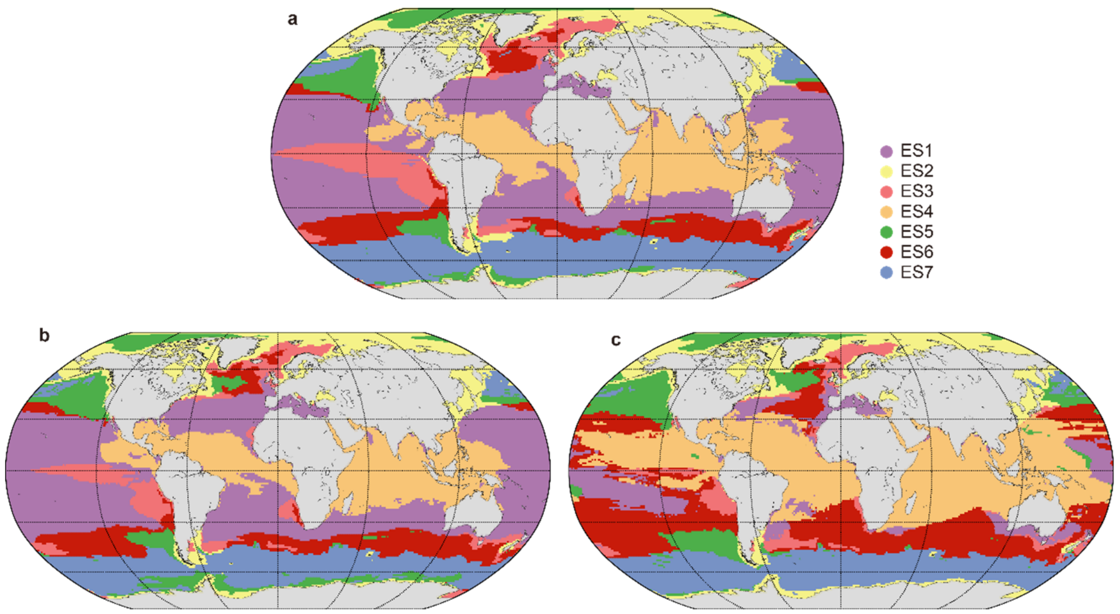

Supplementary Figure 20. Global mapping of ocean ecological statuses for 2100 under different future climate change scenarios. a, Ecological status of the current ocean (2023). Additionally, this figure can be found in Fig. 2b. **b**, Ocean ecological status in 2100 under the SSP1-1.9 scenario. **c**, Ocean ecological status in 2100 under the SSP5-8.5 scenario.

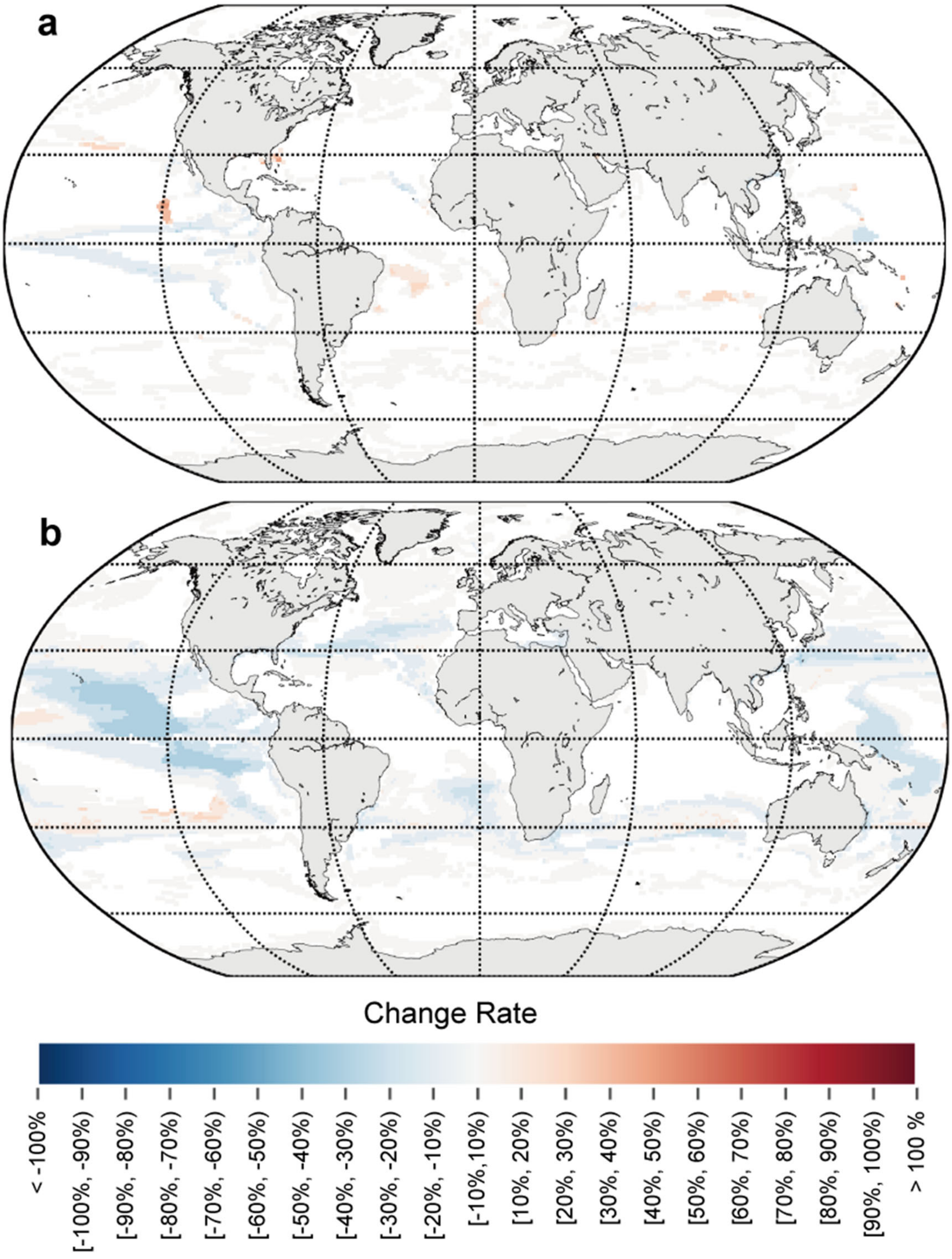

Supplementary Figure 21. Changes in the Shannon indices of ocean microbial communities in 2100 compared to those in 2023 under the SSP1-1.9 (a) and SSP5-8.5 scenarios (b).

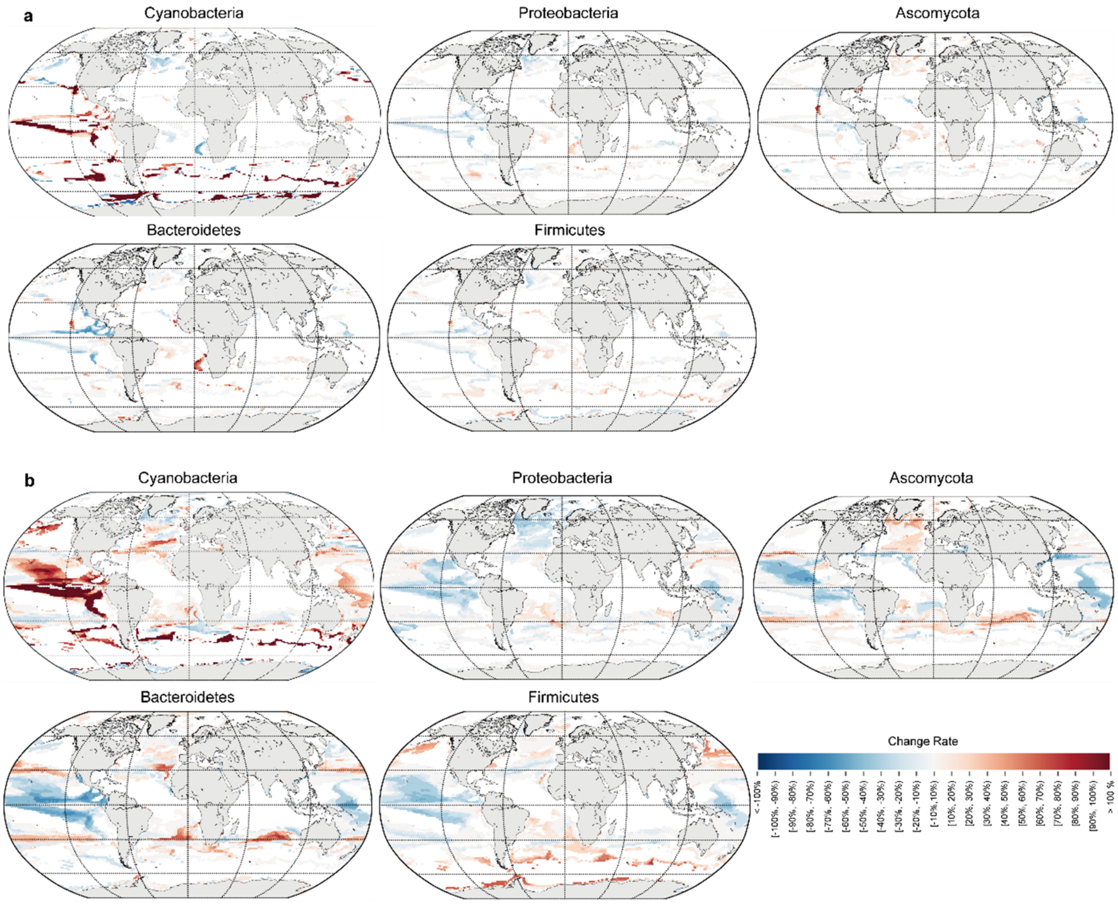

Supplementary Figure 22. Changes in the abundances of dominant taxa in ocean microbial communities in 2100 compared to those in 2023 under the SSP1-1.9 (a) and SSP5-8.5 (b) scenarios.

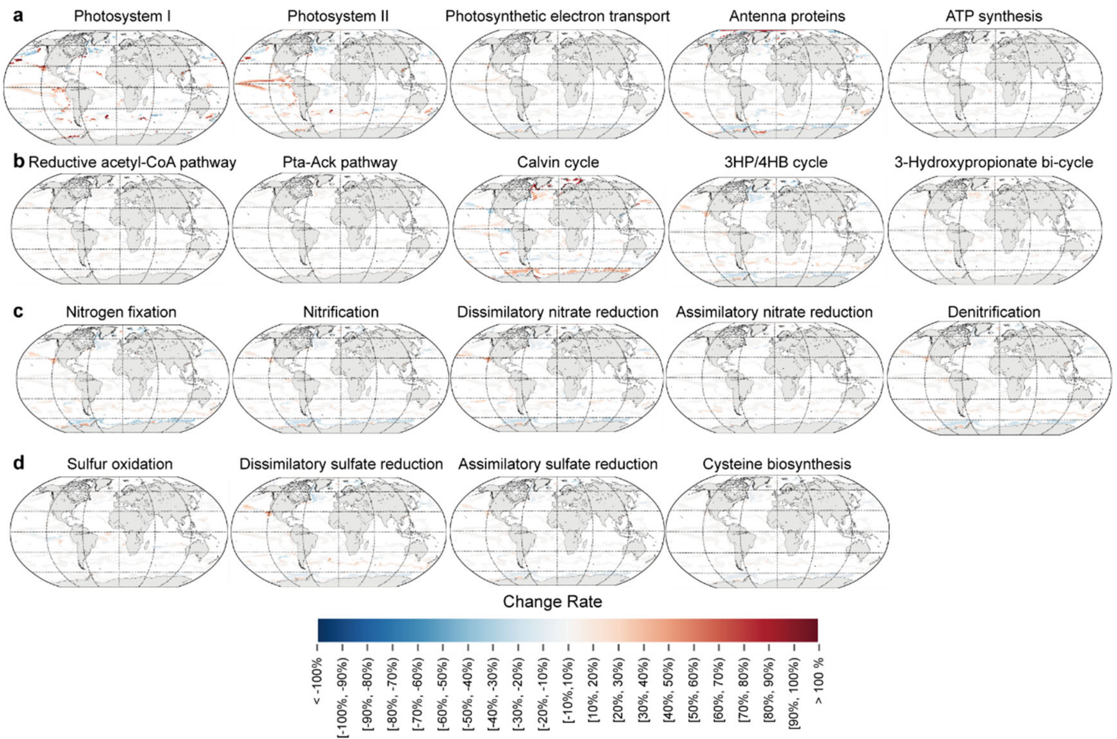

Supplementary Figure 23. Changes in the biogeochemical potentials of ocean microbial communities in 2100 compared to those in 2023 under the SSP1-1.9 scenario. a, Pathways involved in photosynthesis; **b,** pathways involved in carbon fixation; **c,** pathways involved in nitrogen metabolism; **d,** pathways involved in sulfur metabolism. ATP: adenosine triphosphate; Pta-Ack: Phosphate acetyltransferase-acetate kinase; 3HP/4HB: 3-hydroxypropionate/4-hydroxybutyrate.

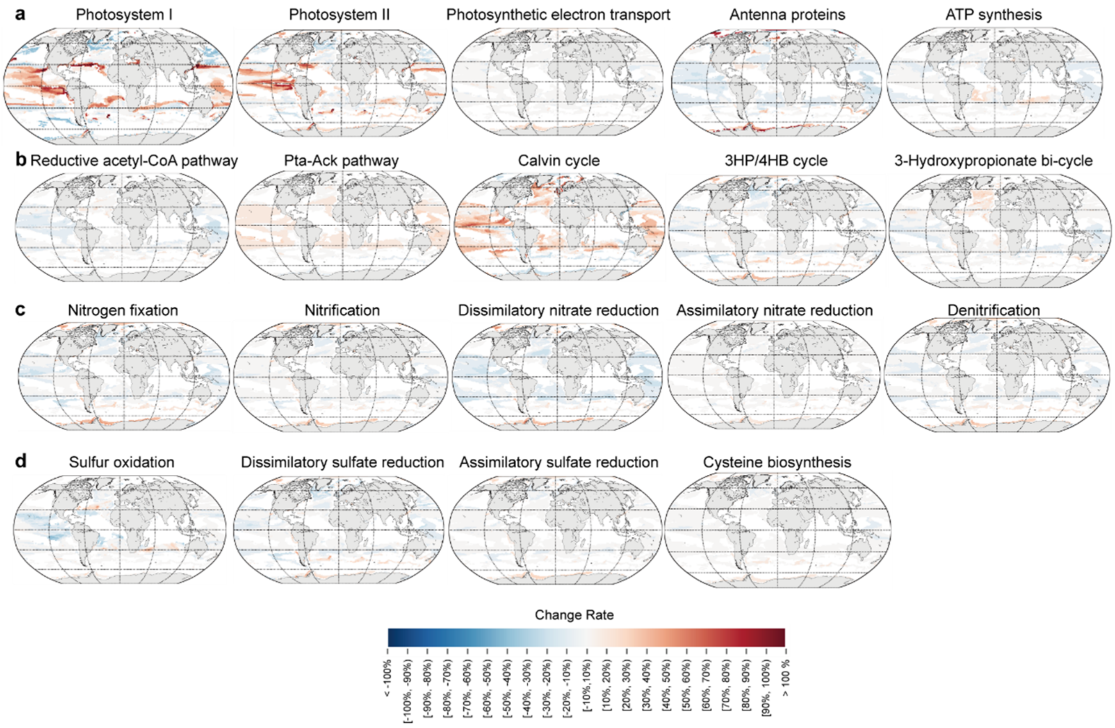

Supplementary Figure 24. Changes in the biogeochemical potentials of ocean microbial communities in 2100 compared to those in 2023 under the SSP5-8.5 scenario. a, Pathways involved in photosynthesis; **b,** pathways involved in carbon fixation; **c,** pathways involved in nitrogen metabolism; **d,** pathways involved in sulfur metabolism. ATP: adenosine triphosphate; Pta-Ack: Phosphate acetyltransferase-acetate kinase; 3HP/4HB: 3-hydroxypropionate/4-hydroxybutyrate.
