# Supplementary material for: Global biogeography of microbes driving ocean ecological status under climate change
Source: Nat Commun. 2024 May 31;15:4657. doi: 10.1038/s41467-024-49124-0 (PMC11143227; doi:10.1038/s41467-024-49124-0)
Supplement: Supplementary file 3 — Description of Additional Supplementary Files [file 41467_2024_49124_MOESM3_ESM.pdf]

Description of Additional Supplementary Files

File Name: Supplementary Data 1
Description: Information of 953 metagenomic samples collected from Bio-GO-SHIP.

File Name: Supplementary Data 2
Description: Information of biogeochemical marker genes.

File Name: Supplementary Data 3
Description: Environmental factors linked to each sample.

File Name: Supplementary Data 4
Description: Dataset for machine learning used to quantitatively predict each microbial index.

File Name: Supplementary Data 5
Description: Machine learning performance in algorithm selection and hyperparameter tuning for regression.

File Name: Supplementary Data 6
Description: Hyperparameter tuning for Hierarchical clustering.

File Name: Supplementary Data 7
Description: Results of Nonparametric test and Cohen's d between each pair of ecological status.

File Name: Supplementary Data 8
Description: Dataset for machine learning classifiers used to predict ecological status

File Name: Supplementary Data 9
Description: Machine learning performance in algorithm selection and hyperparameter tuning for ecological status prediction.
