# Supplementary material for: Global biogeography of microbes driving ocean ecological status under climate change
Source: Nat Commun. 2024 May 31;15:4657. doi: 10.1038/s41467-024-49124-0 (PMC11143227; doi:10.1038/s41467-024-49124-0)
Supplement: Supplementary file 13 — Reporting Summary [file 41467_2024_49124_MOESM13_ESM.pdf]

nature portfolio

Corresponding author(s): Haifeng Qian

Last updated by author(s): May 16, 2024

Reporting Summary

Nature Portfolio wishes to improve the reproducibility of the work that we publish. This form provides structure for consistency and transparency in reporting. For further information on Nature Portfolio policies, see our Editorial Policies and the Editorial Policy Checklist.

Statistics

For all statistical analyses, confirm that the following items are present in the figure legend, table legend, main text, or Methods section.

n/a	Confirmed	
☐	☒	The exact sample size (*n*) for each experimental group/condition, given as a discrete number and unit of measurement
☐	☒	A statement on whether measurements were taken from distinct samples or whether the same sample was measured repeatedly
☐	☒	The statistical test(s) used AND whether they are one- or two-sided<br>*Only common tests should be described solely by name; describe more complex techniques in the Methods section.*
☒	☐	A description of all covariates tested
☐	☒	A description of any assumptions or corrections, such as tests of normality and adjustment for multiple comparisons
☐	☒	A full description of the statistical parameters including central tendency (e.g. means) or other basic estimates (e.g. regression coefficient) AND variation (e.g. standard deviation) or associated estimates of uncertainty (e.g. confidence intervals)
☐	☒	For null hypothesis testing, the test statistic (e.g. *F*, *t*, *r*) with confidence intervals, effect sizes, degrees of freedom and *P* value noted<br>*Give P values as exact values whenever suitable.*
☒	☐	For Bayesian analysis, information on the choice of priors and Markov chain Monte Carlo settings
☒	☐	For hierarchical and complex designs, identification of the appropriate level for tests and full reporting of outcomes
☐	☒	Estimates of effect sizes (e.g. Cohen's *d*, Pearson's *r*), indicating how they were calculated

Our web collection on statistics for biologists contains articles on many of the points above.

Software and code

Policy information about availability of computer code

Data collection	The raw data of metagenomic samples were qualified by FastQC (v0.11.5), and then trimmed and quality-filtered using Trimmomatic (v0.36). Then, biogeochemical marker genes were annotated with BWA (v0.7.13). BWA (v0.7.13) was used for mapping reads , and the unmapped reads were removed using Samtools (v1.3.1). Abundance of genes were calculated by scripts, which have been submitted to GitHub and mentioned in the "Code availability". Taxonomic annotation was performed by Kraken2 (v2.1.2) with the default parameters.
Data analysis	Significant differences were identified using multiple methods. The adonis test with 999 permutations was performed with the 'vegan' R package (v2.5-7) to determine the temporal and spatial variation in both the structure and function of the microbial communities. The Kruskal–Wallis H test with pairwise comparisons (Dunn's test) and Cohen's d calculation were performed with the 'scipy' Python package (v1.13.0) to further evaluate the hierarchical clustering performance. The Friedman test with Nemenyi pairwise comparisons was performed with the 'scipy' Python package (v1.13.0) to determine the different performances of the five machine learning regression algorithms under the best hyperparameter combinations. All these statistical tests were two-sided, and the p-value were adjusted by Bonferroni correction. All the maps were visualized on a world map using the 'Basemap' Python package. Plots of the principal component analysis of ecological status were visualized using the 'matplotlib' Python package. Point plots, density plots, line plots, and box plots were visualized using the 'ggplot2' R package. Heatmaps were constructed using TBtools (v2.0.42). All schematic diagrams and elements in this study were drawn using BioRender (v1.0; https://app.biorender.com) with full publishing rights. All other plots (e.g., pie plots and histograms) were generated using GraphPad Prism (v8.0.2). All R (v3.6.3) scripts were run in Rstudio (v1.4.1103). All machine learning scripts were performed in Python (v3.9) using PyCharm Community Edition (2021.2.2).

For manuscripts utilizing custom algorithms or software that are central to the research but not yet described in published literature, software must be made available to editors and reviewers. We strongly encourage code deposition in a community repository (e.g. GitHub). See the Nature Portfolio guidelines for submitting code & software for further information.

Data

Policy information about availability of data

All manuscripts must include a data availability statement. This statement should provide the following information, where applicable:

- Accession codes, unique identifiers, or web links for publicly available datasets
- A description of any restrictions on data availability
- For clinical datasets or third party data, please ensure that the statement adheres to our policy

The sequence data were collected from Bio-GO-SHIP and are publicly available. Information for all the metadata, including the accession codes, is provided in Supplementary Data 1. The sequences in our database of biogeochemical marker genes are available at https://doi.org/10.6084/m9.figshare.25634544.v1.

Research involving human participants, their data, or biological material

Policy information about studies with human participants or human data. See also policy information about sex, gender (identity/presentation), and sexual orientation and race, ethnicity and racism.

Reporting on sex and gender	Not relevant to this study.
Reporting on race, ethnicity, or other socially relevant groupings	Not relevant to this study.
Population characteristics	Not relevant to this study.
Recruitment	Not relevant to this study.
Ethics oversight	Not relevant to this study.

Note that full information on the approval of the study protocol must also be provided in the manuscript.

Field-specific reporting

Please select the one below that is the best fit for your research. If you are not sure, read the appropriate sections before making your selection.

☐ Life sciences ☐ Behavioural & social sciences ☒ Ecological, evolutionary & environmental sciences

For a reference copy of the document with all sections, see nature.com/documents/nr-reporting-summary-flat.pdf

Ecological, evolutionary & environmental sciences study design

All studies must disclose on these points even when the disclosure is negative.

Study description	In this study, we gathered a substantial dataset of 953 ocean samples from an extensive metagenome sampling project, namely, Bio-GO-SHIP (Fig. 1a and Supplementary Data 1), for taxonomic and functional annotations. For functional annotation, we compiled a database of biogeochemical marker genes associated with the core pathways of photosynthesis, carbon fixation, nitrogen metabolism, and sulfur metabolism (Supplementary Data 2). By using the above dataset and database, we overviewed the variations in microbial communities in oceans and their links to environmental conditions. Subsequently, we constructed machine learning models for each microbial index and predicted their current distribution patterns in the global ocean. In this step, we also established an ecological status, which serves as a composite representation of microbial communities, considering their functional traits, diversities, and structures. Finally, we harnessed machine learning techniques to elucidate how climate change might affect the future alteration of ecological status and to pinpoint the key drivers behind this transformation.
Research sample	We collected 953 metagenome samples of surface oceans from the large-scale metagenome sampling project Bio-GO-SHIP, which was performed utilizing standard pipelines (Fig. 1a and Supplementary Data 1). The raw sequencing data were then downloaded from the NCBI-SRA database. Detailed information on the metagenomes is available in Supplementary Data 1, including the study ID/title, sample ID/title, sequencing strategy, laboratory, organization, isolation source, collection date, latitude, longitude, geological location and Longhurst Province code.
Sampling strategy	In this study, we gathered a substantial dataset of 953 ocean samples from an extensive metagenome sampling project, namely, Bio-GO-SHIP. This project is well-organized and can minimize sample biases.
Data collection	The 953 metagenomic samples were downloaded by ZYZ, QZ, BFC, YTY and NHX from the NCBI-SRA database.
Timing and spatial scale	The 953 metagenomic samples were downloaded in September 2023.
Data exclusions	There was no data exclusion in the analysis of the variations in microbial communities in oceans. However, 63 samples were excluded

Data exclusions	in the analysis related to environmental conditions, as we cannot obtained all environmental variables for these samples.
Reproducibility	All the scripts and codes in this study are available online at https://github.com/ZhenyanZhang/Ecological_status. At the meantime, we provided the accession codes for our 953 samples.
Randomization	Not relevant to this study, as all the data we used were public.
Blinding	Not relevant to this study, as all the data we used were public.

Did the study involve field work? ☐ Yes ☒ No

Reporting for specific materials, systems and methods

We require information from authors about some types of materials, experimental systems and methods used in many studies. Here, indicate whether each material, system or method listed is relevant to your study. If you are not sure if a list item applies to your research, read the appropriate section before selecting a response.

Materials & experimental systems

n/a	Involved in the study
☒	☐ Antibodies
☒	☐ Eukaryotic cell lines
☒	☐ Palaeontology and archaeology
☒	☐ Animals and other organisms
☒	☐ Clinical data
☒	☐ Dual use research of concern
☒	☐ Plants

Methods

n/a	Involved in the study
☒	☐ ChIP-seq
☒	☐ Flow cytometry
☒	☐ MRI-based neuroimaging

Plants

Seed stocks	*Report on the source of all seed stocks or other plant material used. If applicable, state the seed stock centre and catalogue number. If plant specimens were collected from the field, describe the collection location, date and sampling procedures.*
Novel plant genotypes	*Describe the methods by which all novel plant genotypes were produced. This includes those generated by transgenic approaches, gene editing, chemical/radiation-based mutagenesis and hybridization. For transgenic lines, describe the transformation method, the number of independent lines analyzed and the generation upon which experiments were performed. For gene-edited lines, describe the editor used, the endogenous sequence targeted for editing, the targeting guide RNA sequence (if applicable) and how the editor was applied.*
Authentication	*Describe any authentication procedures for each seed stock used or novel genotype generated. Describe any experiments used to assess the effect of a mutation and, where applicable, how potential secondary effects (e.g. second site T-DNA insertions, mosiacism, off-target gene editing) were examined.*
